# Role of Bile-Derived Extracellular Vesicles in Hepatocellular Proliferation after Partial Hepatectomy in Rats

**DOI:** 10.3390/ijms24119230

**Published:** 2023-05-25

**Authors:** Shinpei Sugahara, Hiroaki Haga, Chisaki Ikeda, Naohiko Makino, Akiko Matsuda, Yasuharu Kakizaki, Kyoko Hoshikawa, Tomohiro Katsumi, Tetsuya Ishizawa, Toshikazu Kobayashi, Keita Maki, Fumiya Suzuki, Ryoko Murakami, Hidenori Sato, Yoshiyuki Ueno

**Affiliations:** 1 Department of Gastroenterology, Faculty of Medicine, Yamagata University, 2-2-2 Iidanishi, Yamagata 990-8595, Japan; 2 Yamagata University Health Administration Center, 1-4-12 Kojirakawa-Machi, Yamagata 990-8560, Japan; 3 Genomic Information Analysis Unit, Department of Genomic Cohort Research, Faculty of Medicine, Yamagata University, 2-2-2 Iidanishi, Yamagata 990-8595, Japan

**Keywords:** liver regeneration, extracellular vesicle, bile, hepatocyte, cell cycle

## Abstract

Although liver regeneration has been extensively studied, the effects of bile-derived extracellular vesicles (bile EVs) on hepatocytes has not been elucidated. We examined the influence of bile EVs, collected from a rat model of 70% partial hepatectomy (PH), on hepatocytes. We produced bile-duct-cannulated rats. Bile was collected over time through an extracorporeal bile duct cannulation tube. Bile EVs were extracted via size exclusion chromatography. The number of EVs released into the bile per liver weight 12 h after PH significantly increased. Bile EVs collected 12 and 24 h post-PH, and after sham surgery (PH12-EVs, PH24-EVs, sham-EVs) were added to the rat hepatocyte cell line, and 24 h later, RNA was extracted and transcriptome analysis performed. The analysis revealed that more upregulated/downregulated genes were observed in the group with PH24-EVs. Moreover, the gene ontology (GO) analysis focusing on the cell cycle revealed an upregulation of 28 types of genes in the PH-24 group, including genes that promote cell cycle progression, compared to the sham group. PH24-EVs induced hepatocyte proliferation in a dose-dependent manner in vitro, whereas sham-Evs showed no significant difference compared to the controls. This study revealed that post-PH bile Evs promote the proliferation of the hepatocytes, and genes promoting cell cycles are upregulated in hepatocytes.

## 1. Introduction

The liver is an organ with an extremely high regenerative capacity. The rat liver, in particular, is restored to its original size in approximately 1 week, even if 70% of the liver has been resected [1]. The constituent cells of a damaged liver are hypertrophied and hyperplastic, and continuously increase and restore the volume of the organ [2]. For some time now, studies focusing on growth factors have been actively conducted with the aim to elucidate the regenerative mechanism. Liver regeneration is a complex process involving several types of cells, such as endothelial cells, liver stem cells, and mesenchymal stem cells; this process is strictly regulated by the cytokine network including the hepatocyte growth factor (HGF), the epidermal growth factor (EGF), and the transforming growth factor (TGF-β1) [3]. Although it is still under debate, liver stem cells are believed to be present near the contact point between hepatocytes and interlobular bile ducts (canals of Hering), and these are activated during hepatic disorders, forming oval cells to regenerate the liver [4]. Blood vessels (portal veins, arteries, and veins) and bile ducts are distributed in the liver. Owing to the fact that bile flows in the canals of Hering, bile is thought to have some influence on liver regeneration. In addition, after being secreted from bile ducts to the duodenum, biomolecules and metabolites in bile are reabsorbed at the end of the ileum, return to the liver via portal veins, and are secreted into bile again [5,6,7]. Approximately 95% of bile acid, the main component of bile, is reabsorbed by this enterohepatic circulation; this step ensures the efficient use of bile [8]. However, research on the relationship between bile and liver regeneration is lacking. Studies have only reported that bile flow rate per liver weight increases after 70% partial hepatectomy (PH) [9], that Cyp7a1—the rate-limiting enzyme of bile acid production—is rapidly suppressed during liver regeneration [10], and that bile acid acts as the signal that controls the start and end of liver regeneration [11].

Extracellular vesicle (EV) is the general term for vesicles, such as exosomes, that are approximately 50–150 nm in size and microvesicles of a wide range of sizes from 100 to 1000 nm. However, in recent years, studies have clarified that these EVs contain nucleic acids and proteins, and are involved in various vital phenomena by transferring these molecules between cells [12,13,14]. EVs are reportedly present in bile, and bile EVs are released from hepatocytes to bile canaliculi [15]. In recent years, the number of studies on the relationship between liver generation and EVs has gradually increased. However, these studies only reported that EVs are a factor promoting liver regeneration; some of the findings reported in these studies are as follows: hepatocyte-derived EVs transport the mechanism for sphingosine-1-phosphate synthesis, thereby promoting liver regeneration [16]; and miR-10a may increase in peripheral blood EVs after rat PH, promoting liver regeneration [17]. However, several unclear elements exist regarding the involvement of EVs in liver regeneration. Furthermore, the abovementioned reports examined EVs in peripheral blood, and no study has reported on EVs in bile and liver regeneration thus far. This study aimed to investigate the effects on hepatocytes in vitro using bile EVs during liver regeneration in a rat 70% PH model.

## 2. Results

### 2.1. Extraction of Rat Bile-Derived EVs

**Figure 1:**
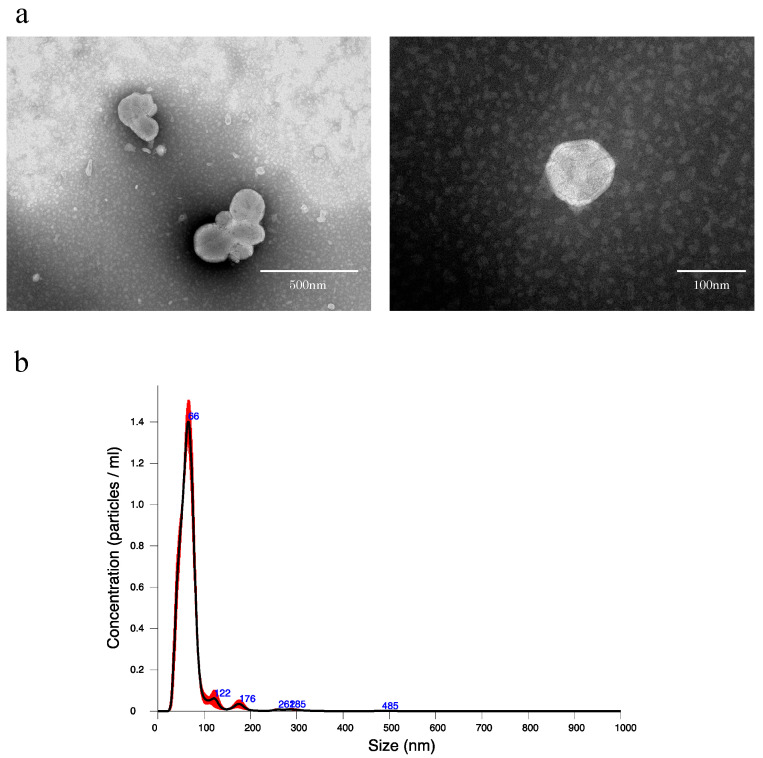
(**a**) Transmission electron microscope images of extracellular vesicles derived from normal rat bile. (**b**) Nanoparticle tracking analysis of extracellular vesicles derived from normal rat bile. An amount of 1.5 mL of EV extract was obtained from 500 μL of bile by qEV, and the EV extract was diluted 20-fold in PBS and measured. The black line indicates the average size/concentration, while the red error bars represent ± 1 standard error of the mean.

To confirm that EVs can be extracted from rat bile, using the qEV original (35 nm) extracellular vesicle extraction kit, EVs were extracted from normal rat bile and verified. When the collected EV extract was observed by transmission electron microscope (TEM), many round vesicles 40–100 nm in diameter with lipid bilayer membrane structure could be confirmed (Figure 1a). In addition, when the EV extract was verified by nanoparticle tracking analysis (NTA), the mean particle size was 69.3 ± 2.0 nm, while the mean number of particles in bile was 5.39 × 10^10^ ± 2.00 × 10^9^ particles/mL (Figure 1b).

### 2.2. Examination of the Number of EVs

**Figure 2:**
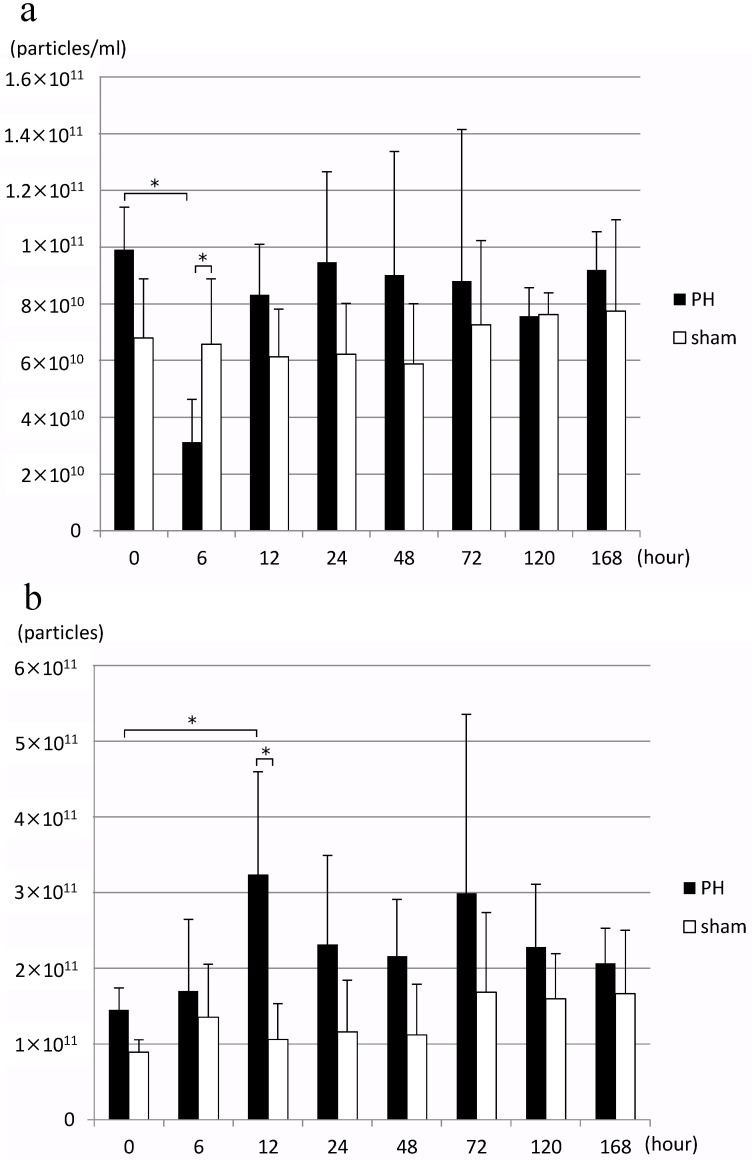
(**a**) Changes in the concentration of rat bile-derived extracellular vesicles over time (particles/mL). (**b**) Changes in the number of extracellular vesicles released into rat bile over time (particles/liver weight/hour) PH: 70% partial hepatectomy, sham: sham surgery, *n* = 5 mice/group, *p* < 0.05 (*).

In the PH group, EV concentration in bile decreased significantly 6 h after PH compared to before surgery, but it returned to an EV concentration equivalent to that before surgery after 12 h (Figure 2a). When the number of EVs in bile released per liver gram per hour, bile outflow (mL/h), and estimated liver weight were calculated from this result, the number of EVs significantly increased 12 h after PH compared to before surgery (Figure 2b). Estimated liver weight was calculated from the rate of increase in the weight of residual liver over time as reported by Higgins and Anderson [1], and resected liver weight. However, no significant change was observed in the number of EVs of the sham surgery group.

### 2.3. Verification of Bile EV Migration to Hepatocytes

**Figure 3:**
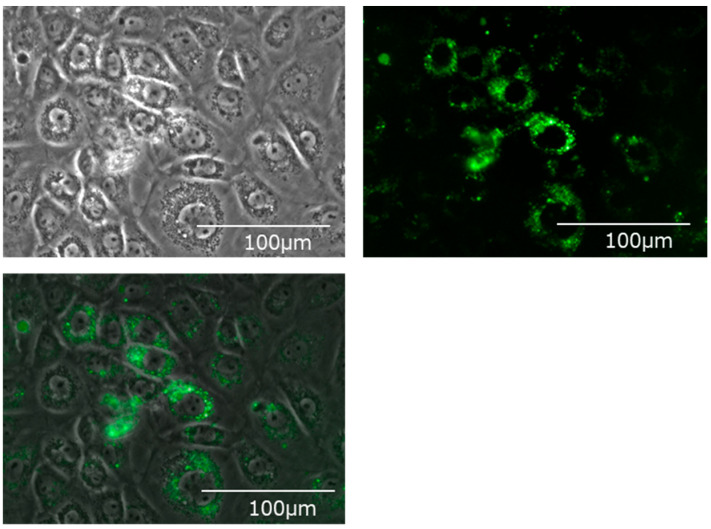
Bile-derived extracellular vesicles incorporated into the cytoplasm of the hepatic cell line (BRL-3A). Upper left: A phase contrast microscope image. Upper right: A fluorescence microscope image. Mem Dye-Deep Green: Ex 488 nm/Em 490–540 nm. Bottom: An overlay image.

Fluorescently labeled EVs were added, cultured for 24 h, and the hepatocyte cell line (BRL-3A) was observed with a fluorescence microscope. Many fluorescently labeled EVs incorporated into the cytoplasm of hepatocytes could be confirmed (Figure 3).

### 2.4. Transcriptome Analysis

**Figure 4:**
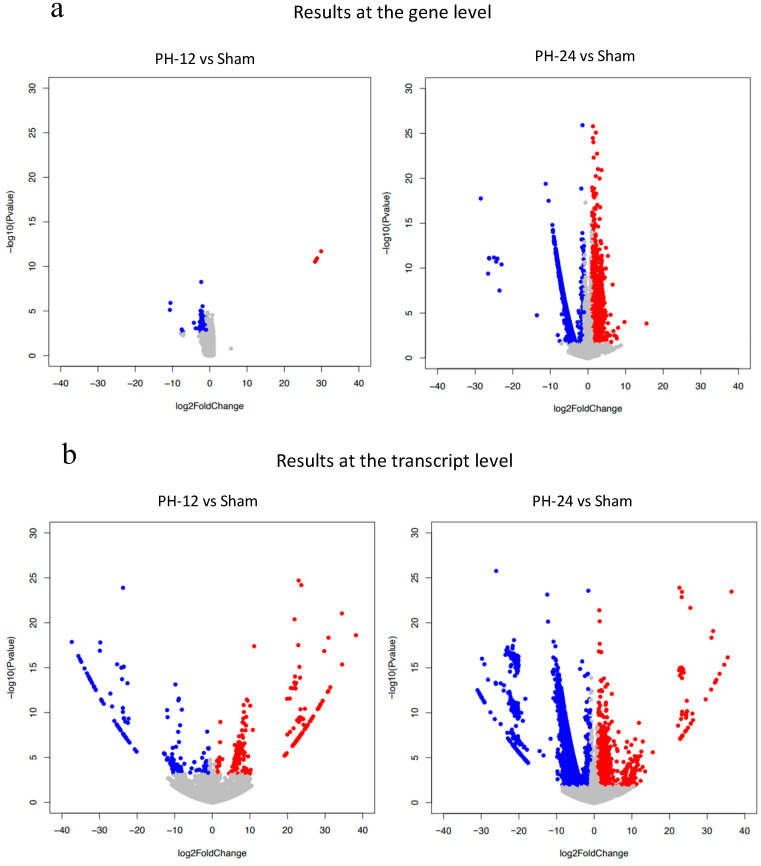
Volcano plot show the number of upregulated or downregulated genes in PH-12 group and PH-24 group compared to the sham group. Red dots shows upregulation (log2 Fold change > 1, adjusted *p* value < 0.05). Blue dots shows downregulation (log2 Fold change < −1, adjusted *p* value < 0.05). all others are gray dots. (**a**) Comparison at the gene level. (**b**) Comparison at the transcript level.

**Figure 5:**
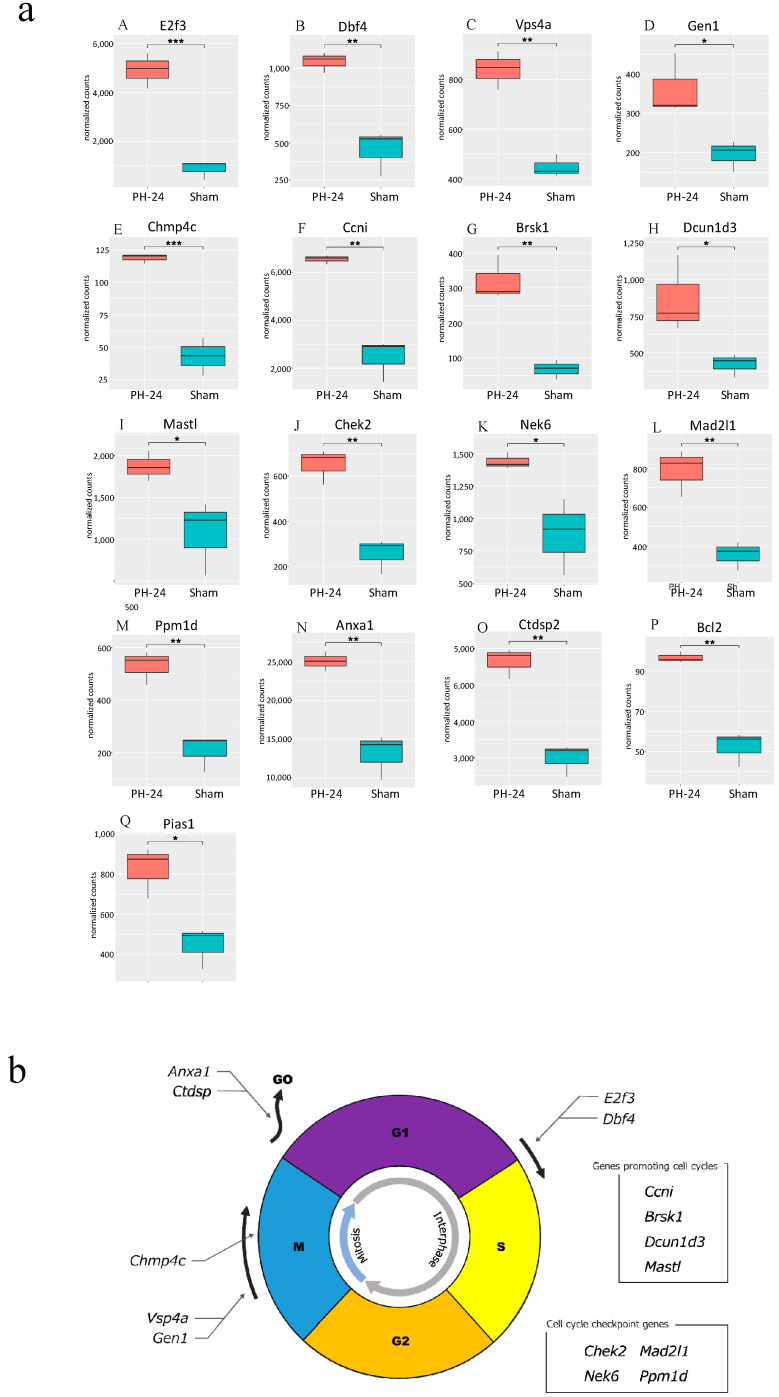
(**a**) Comparison between the PH-24 group and the sham group for genes reported to be involved in cell cycles. (**A**,**B**): Genes promoting the G1/S migration (*E2f3*, *Dbf4*). (**C**,**D**): Genes controlling the centrosome and promoting mitosis in the M phase (*Vsp4a*, *GEN1*). (**E**) A gene promoting accurate chromosome segregation in the late M phase (*Chmp4c*). (**F**) A gene constantly acting on and promoting cell cycles (*Ccni*). (**G**–**I**) Genes whose specific functions are unclear, but are considered to promote cell cycles (*Brsk1*, *Dcun1d3*, *Mastl*). (**J**–**M**) Cell cycle checkpoint genes (*Chek2*, *Nek6*, *Mad2l1*, *Ppm1d*). (**N**,**O**) Genes halting cell cycles (transitioning from G1 phase to G0 phase) (*Anxa1*, *Ctdsp2*). (**P**) A gene demonstrating the proliferative effect at the initial stage of liver regeneration (*Bcl2*). (**Q**) A gene promoting the induction of the bile acid receptor in the hepatocyte nucleus (*Pias1*). *p* < 0.05 (*), *p* < 0.01 (**), *p* < 0.001 (***). (**b**) Upregulated genes influencing cell cycles in the PH-24 group. PH: 70% partial hepatectomy.

Bile EVs collected 12 and 24 h post-PH, and after sham surgery (PH12-EVs, PH24-EVs, sham-EVs) were added to the rat hepatocyte cell line, and 24 h later, RNA was extracted and transcriptome analysis performed. When the PH-12 group (hepatocyte cell line added with PH12-EVs) and the sham group (hepatocyte cell line added with sham-EVs) were compared, four types of genes were upregulated in the PH-12 group, while 103 types of genes were downregulated. However, in the comparison between the PH-24 group (hepatocyte cell line added with PH24-EVs) and the sham group, 926 types of genes were upregulated and 2036 types of genes were downregulated in the PH-24 group; and more changes in genes were observed in the PH-24 group than in the PH-12 group (Figure 4). Therefore, using the expression data of the PH-24 group and the sham group compared with the control group, GO enrichment analysis was performed. When the conditions were set as containing “cell cycle” in Term and *p* value < 0.01 in order to narrow down the obtained results, no GO term matching the conditions was obtained in the sham group, but three types of GO terms were obtained in the PH-24 group (Table 1). Out of the 122 genes (ID: GO:0044772, Term: mitotic cell cycle phase transition), 133 genes (ID: GO:0044770, Term: cell cycle phase transition) and 67 genes (ID: GO:0044843, Term: cell cycle G1/S phase transition) matching these three types of GO Terms, when log_2_ fold change was compared between the PH-24 group and the sham group using the 133 genes excluding repetitions, 28 types of genes were significantly upregulated in the PH-24 group (Table 2). They included multiple genes that influence cell cycles, such as the genes that have been reported to promote cell cycles, such as *E2f3*, *Dbf4*, *Vsp4a*, *Gen1*, and *Chmp4c*, and cell cycle checkpoint genes, such as *Chek2*, *Nek6*, *Mad2l1*, and *Ppm1d* (Figure 5a(A–O)). On the other hand, even though 19 types of genes were downregulated, they did not include noteworthy genes involved in cell cycles.

### 2.5. Examination of Cell Proliferative Ability

**Figure 6:**
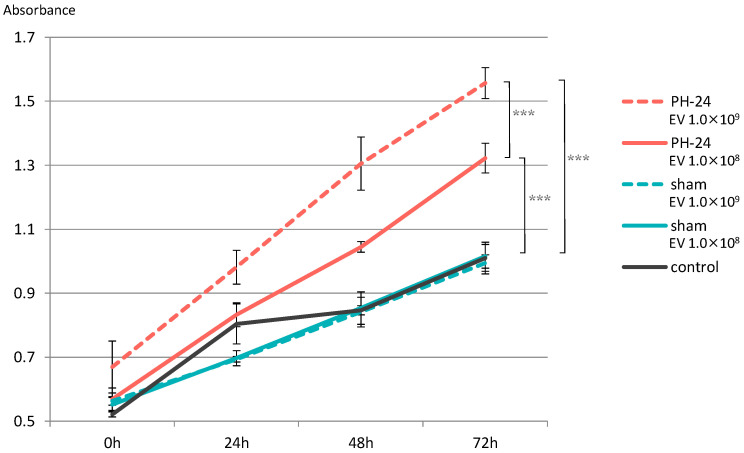
EVs were added to the hepatocyte cell line (BRL-3A), and proliferative ability was verified by the MTS assay. Proliferation was significantly promoted in the PH-24 group than in the sham group and the control group. In addition, in the PH-24 group, proliferation was significantly promoted in the group with EVs added at 1.0 × 10^9^ particles/well (dashed line) than in the group with EVs added at 1.0 × 10^8^ particles/well (solid line). No difference was observed in the sham group and the control group. PH: 70% partial hepatectomy *p* < 0.001 (***).

Proliferation of hepatocyte cell line significantly increased in both the group with EVs added at 1.0 × 10^8^ particles/well and the group with EVs added at 1.0 × 10^9^ particles/well in the PH-24 group, compared to the sham group and the control group. Furthermore, looking at the PH-24 group, it could be confirmed that the group with EVs added at 1.0 × 10^9^ particles/well significantly proliferated more than the group with EVs added at 1.0 × 10^8^ particles/well. However, no significant difference was observed in the sham group and the control group (Figure 6).

## 3. Discussion

For some time now, the liver has been known to possess an exceptionally high regenerative capacity among organs. To date, many factors involved in liver regeneration have been reported; however, the detailed mechanisms are unknown. By approaching liver regeneration from a new perspective, namely EVs in bile, this study demonstrated for the first time that ① the number of EVs released from hepatocytes into bile after 70% partial hepatectomy increases, ② EVs incorporated into hepatocytes may upregulate genes in hepatocytes, activating cell cycles, and ③ EVs in bile after hepatectomy promote the proliferation of hepatocytes.

Reports on the relationship between bile and liver regeneration to date have included a report that bile flow rate per liver weight increases after PH [9], that Cyp7a1, the rate-limiting enzyme of bile acid production, is rapidly suppressed during liver regeneration [10], and that bile acid acts as the signal during liver regeneration to control the start and end of liver regeneration [11]. However, with respect to EVs and liver regeneration, it has been reported that hepatocyte-derived EVs transport the mechanism for sphingosine-1-phosphate synthesis, promoting liver regeneration [16], and that miR-10a may increase in peripheral blood EVs after rat PH, promoting liver regeneration [17]. However, they are both studies on EVs in peripheral blood, and there has been no report on EVs in bile and liver regeneration so far. In this study, we focused on bile-derived EVs, and examined the roles of bile EVs in liver regeneration.

In addition, previous reports on bile with animal experiment models, such as rats, involved bile collection after laparotomy, and euthanasia after collection was completed. In other words, they could not examine how bile in the same individual changes over time. This study is thus also the first study to overcome this by producing bile-duct-cannulated rats.

The standard method to extract EVs is ultracentrifugation, but EV samples may be lost in the extraction process, and that is a problem. In the present study, as the amount of bile collected from rats was small (approximately 1 mL), size exclusion chromatography, which enabled more efficient extraction, was used. First, as there had been no report on the extraction of EVs from bile with size exclusion chromatography using qEV, we confirmed if EVs could be extracted by the same method as blood samples. When extraction was performed according to the protocol and observed by TEM, many various vesicles 40–100 nm in size presenting lipid bilayer membrane structure were confirmed (Figure 1a). The analysis by NAT also revealed that particle size peaked at approximately 70 nm, and almost no coarse particles considered to be contaminants were observed (Figure 1b). Based on the above, it was determined that rat bile-derived EVs can be extracted using qEV.

Next, we examined how the number of bile EVs after PH changes over time using NTA. Even though EV concentration temporarily decreased as the total liver weight decreased immediately after PH, it restored the EV concentration equivalent to preoperative levels after 12 h (Figure 2a). Similar to a previous report [9], bile flow rate per liver weight in this study increased after PH, and looking at the number of EVs released per liver weight, the number of EVs released did not decrease even after PH, but increased significantly after 12 h (Figure 2b). That is, EVs released into bile increase with liver regeneration and peak after 12 h.

Subsequently, we verified how bile EVs influence hepatocytes. First, to confirm that bile EVs are incorporated into hepatocytes, fluorescently labeled rat bile-derived EVs were added to the rat hepatocyte cell line (BRL-3A) cultured in vitro, and cultured. When observed with a fluorescence microscope, it could be confirmed that many EVs had been incorporated into the cytoplasm (Figure 3). In order to clarify how these EVs incorporated into hepatocytes act, we carried out further verification. The hepatocyte cell line (BRL-3A) was cultured, PH12-EVs, PH24-EVs and sham-EVs were added respectively and cultured for 24 h, cells were collected, RNAs were extracted, and verification was performed by next-generation sequencing; it was found that the number of upregulated or downregulated genes 24 h after PH increased (Figure 4). When examining with a focus on cell cycles in particular, out of the significantly upregulated genes in the PH-24 group compared to the sham group, genes such as *E2f3* [18,19] and *Dbf4* [20], which promote the G1/S transition (Figure 5a(A,B)), *Vsp4a* [21] and *Gen1* [22], which control the centrosome and promote mitosis in the M phase (Figure 5a(C,D)), *Chmp4c* [23], which promotes accurate chromosome segregation in the late M phase (Figure 5a(E)), *Ccni* [24], which constantly promotes cell cycles (Figure 5a(F)), and *Brsk1* [25], *Dcun1d3* [26] and *Mastl* [27], whose specific functions are unclear, but are considered to promote cell cycles, were upregulated (Figure 5a(G–I)). Furthermore, cell cycle checkpoint genes such as *Chek2* [28,29], *Nek6* [30,31], *Mad2l1* [32], *Ppm1d* [33,34,35] required for proper cell division progression were also upregulated (Figure 5a(J–M)). However, genes that stop cell cycles (*Anxa1* [36], *Ctdsp2* [37]) were also upregulated (Figure 5a(N,O)). PH24-EVs are considered to promote cell cycles of hepatocytes while controlling accurate cell division (Figure 5b).

Moreover, *Bcl2* [38] believed to induce the proliferative promotion effect at the initial stage of liver regeneration was upregulated (Figure 5a(P)). In addition, *Pias1* [39], that induces the bile acid receptor (Farnesoid X receptor: FXR) in the hepatocyte nucleus was also upregulated (Figure 5a(Q)). A previous report has stated that bile acid exposure activates FXR, initiating liver regeneration, while decreased bile acid exposure level halts liver regeneration [11]. It is believed that the upregulation of these genes promotes liver regeneration itself.

Based on this result, when the proliferative ability of PH24-EVs was verified in vitro, it was clarified that, compared to the sham group and the control group, both the group with EVs added at 1.0 × 10^8^ particles/well and the group with EVs added at 1.0 × 10^9^ particles/well of the group with PH24-EVs added, statistically significantly promote proliferation, and PH24-EVs promote the proliferation of hepatocytes in a concentration-dependent manner (Figure 6). Considering that no significant difference was observed in the sham group and the control group, PH24-EVs, rather than bile EVs themselves, likely promote the proliferation of hepatocytes.

Bile EVs used for verification in this study were extracted from the bile flowing out of rat extrahepatic bile ducts. We demonstrated that PH24-EVs promote the proliferation of hepatocytes in a concentration-dependent manner, but the two following pathways of EVs that have been released from hepatocytes into bile to be incorporated into hepatocytes again are possible. First, between intrahepatic bile canaliculi and interlobular bile ducts, EVs may be transferred between hepatocytes. Second, they may temporarily flow out into the intestinal tract with bile, be reabsorbed at the end of the ileum, and incorporated into hepatocytes again via the enterohepatic circulation. In the former case, bile EVs that increase after PH may be lost without being incorporated into hepatocytes, which is inefficient, so they probably use the pathway via the enterohepatic circulation, or possibly both pathways. However, at present there is no means to collect bile between bile canaliculi and interlobular bile ducts, so it is difficult to verify this.

In addition, the bile used in this study flows out from extrahepatic bile ducts, as mentioned above, so EVs extracted from here are thought to be a mixture of hepatocyte-derived EVs and bile duct cell-derived EVs. The origin of the PH24-EVs promoting the proliferation of hepatocytes could not be determined in the present study. If the bile between bile canaliculi and interlobular bile ducts can be collected, it may be possible to separate hepatocyte-derived EVs and bile duct cell-derived EVs, but at present there is no such method, and it is therefore not feasible.

The limitations of this study include the small sample size, that proteome analysis was not performed, and that the RNA expression and protein expression were not compared. In addition, even though we attempted to analyze proteins and RNAs in EVs, the number of specimens was small, so it was not possible. Furthermore, this study is an in vitro study, so it was not possible to verify how bile EVs influence hepatocytes in vivo. In order to further verify the results of this study, it is considered necessary to compare transcriptome analysis and proteome analysis, as well as to increase the amount of bile collected, analyze the nucleic acids and proteins in EVs, and perform in vivo tests.

## 4. Materials and Methods

### 4.1. Preparation of the Bile Duct Cannulated Rat Model

**Figure 7:**
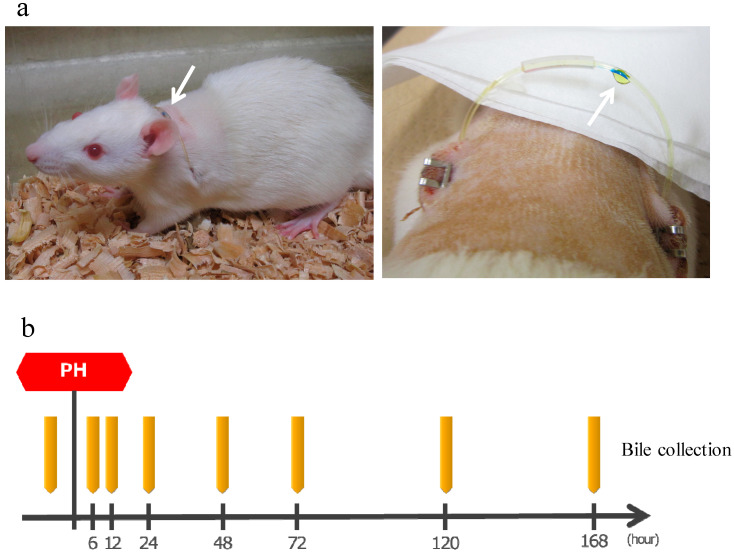
(**a**) **Left**: A bile duct cannulated rat. Arrow shows a catheter guided to the outside of the body. Bile is flowing inside the catheter from the left back toward the right back. **Right**: Bile dripping from a catheter. (**b**) The protocol of bile collection. The rats were bred while supplementing normal saline solution as appropriate. PH: 70% partial hepatectomy.

After making an incision in the abdomen of SD rats (8-weeks old, male), the bile duct was separated, one side of the catheter was inserted into the bile duct on the liver side and fixed, a subcutaneous tunnel was formed, the catheter was guided to the outside of the body through the left back, a loop was formed at the neck, guided into the abdominal cavity via the subcutaneous tunnel from the right back, the tip of the other side was placed in the duodenum, and then the abdomen was closed. This allows convenient bile collection at any time while bile returns into the body when bile collection is not necessary, thereby allowing long-term survival (Figure 7a). The production of bile duct cannulated rats was entrusted to Charles River Laboratories Japan, Inc. (Yokohama, Japan).

### 4.2. Bile Collection 70% Partial Hepatectomy

The bile duct cannulated rats were divided into the two groups of the PH group (*n* = 5) and the sham surgery group (*n* = 5). Furthermore, 70% PH was performed on the PH group according to the methods established by Higgins and Anderson, whereas sham surgery (placebo surgery) was performed on the sham surgery group [1]. First, the bile duct cannulated rats were anesthetized by subcutaneously injecting 0.25 mL of three types of mixed anesthetic agents per 100 g of weight. Next, an incision was made to the abdomen after shaving and disinfection, and the liver to be resected was exposed to the outside of the body. After treating the connective tissues around the liver, the base of the hepatic lobe to be resected was ligated with 2-0 silk suture, and excised with scissors. Afterwards, the abdomen was closed with 6-0 PDS suture. Similarly, in the sham surgery, an incision was made to the abdomen and the liver was exposed to the outside of the body, and then the liver was returned into the body and the abdomen was closed.

After the surgery, bile was collected by the catheter guided to the outside of the body. Approximately 1.0 mL of bile was collected before surgery and 6, 12, 24, 48, 72, 120, and 168 h after surgery and stored at 4 °C (Figure 7b). Rats were supplemented with normal saline solution as appropriate as bile was lost.

In addition, during surgery and bile collection, the three types of mixed anesthetic agents of Yamagata University (medetomidine hydrochloride, midazolam, butorphanol tartrate), which had been improved based on the three types of mixed anesthetic agents reported by Osaka University [40], were used, and atipamezole, which is a medetomidine antagonist, was administered after the completion of surgery.

### 4.3. EV Extraction from Bile

EVs were rapidly extracted from bile according to the protocol using a qEV original (35 nm) extracellular vesicle extraction kit (Izon Science Ltd., Christchurch, New Zealand) [41]. qEV used size exclusion chromatography, and PBS was used for extraction. An amount of 500 μL of bile was placed into the qEV column, and 2.5 mL of PBS was added. After dripping all of the PBS in the column, 500 μL of PBS was added, and the dripped EV extract was collected. This was repeated three times. An amount of 1.5 mL of EV extract was obtained from 500 μL of bile. The EV extract was stored at −80 °C until use.

### 4.4. Evaluation of EVs

#### 4.4.1. Transmission Electron Microscope (TEM)

To observe the EVs with an electron microscope, the obtained EV extract was adsorbed to carbon-coated copper grids (400 mesh), and stained with 2% phosphotungstic acid solution (pH 7.0) for 30 s. Next, we used a transmission electron microscope (JET-1400Plus, JEOL, Ltd., Tokyo, Japan) under an accelerating voltage of 100 kV. Digital images (3296 × 2472 pixels) were captured with a CCD camera (EM-14830RUBY2, JEOL, Ltd., Tokyo, Japan).

#### 4.4.2. Nano Tracking Analysis (NTA)

The size and number of nanoparticles of the obtained EV extract were measured using a nanoparticle analysis system (NanoSight NS300, Malvern Panalytical, Malvern, UK). The EV extract was diluted 20-fold in PBS and used, the tracking of the Brownian motion of nanoparticles in the solution was recorded for 60 s three times, and particle size and number were measured by the NTA 3.1 software.

### 4.5. Verification of Bile EV Migration to Hepatocytes

The cell line derived from normal rat hepatocyte cell line (BRL-3A), obtained from JCRB Cell Bank (Japanese Collection of Research Bioresources Cell Bank) (Osaka, Japan), was inoculated to a 6-well plate (1 × 10^5^ cells/well), and cultured under 5% CO_2_ at 37 °C for 24 h. DMEM, 10% FBS, 1% PS were used as the medium. Rat bile EVs obtained using qEV were fluorescently labeled according to the protocol using the EV membrane fluorescent staining kit (ExoSparkler, Dojindo, Kumamoto, Japan). The EV extract was suspended with PBS to EV 1.0 × 10^10^ particles/100 μL; 2 μL of Mem Dye stock solution was added and stirred. It was incubated at 37 °C for 30 min, placed into a filtration tube and centrifuged at 20–25 °C at 3000 g for 5 min. An amount of 100 μL of PBS was added, centrifuged at room temperature at 3000 g for 5 min, and repeated twice. An amount of 50 μL of PBS was added, then pipetted, and stained EVs were collected. After removing the medium of the cultured cells and washing twice with PBS, exosome-free medium (DMEM, 10% exosome-depleted FBS, 1% PS) was added, 50 μL of stained EVs collected was added, and cultured under 5% CO_2_ at 37℃ for 24 h. The culture supernatant was removed, washed twice with PBS, and a new medium added, then observed with a fluorescence microscope (BZ-X710, Keyence, Osaka, Japan).

### 4.6. Transcriptome Analysis of Hepatocytes after Bile EV Addition

#### 4.6.1. Addition of EVs to the Hepatocyte Cell Line

EVs were extracted from bile 12 and 24 h after PH, and after sham surgery. The rat cell line (BRL-3A) inoculated to a 6-well plate at 1 × 10^5^cells/well was cultured under 5% CO_2_ at 37 °C for 24 h (medium: DMEM, 10% FBS, 1% PS). After removing the medium and washing once with PBS, exosome-free medium (DMEM, 10% exosome-depleted FBS, 1% PS) was added and then divided into four groups: the PH-12 group, the PH-24 group, the sham group and the control group (*n* = 3, respectively). EV extracts 12 and 24 h after PH, as well as after sham surgery (PH12-EVs, PH24-EVs, and sham-EVs, respectively) were added to each group respectively at 1.0 × 10^10^ particles/well, while an extract without EVs (PBS) was added to the control group. Subsequently, they were cultured under 5% CO_2_ at 37 °C for 24 h.

#### 4.6.2. RNA Extraction of Cultured Cells

The culture medium was aspirated and washed with PBS, and the attached cells were treated with trypsin. After exfoliating the cells, medium was added and centrifuged, and the supernatant was aspirated and removed. The pelleted cells were crushed by adding 350 μL of Buffer RLT Plus, and then applied to the spin column and centrifuged. Furthermore, they were applied to the gDNA Eliminator spin column and centrifuged, the flow-through liquid was collected, and 350 μL of 70% ethanol was added and mixed. This was applied to the RNeasy spin column and centrifuged. Moreover, after washing the RNeasy spin column by applying and centrifuging with 700 μL of Buffer RW1 once and 500 μL of Buffer RPE twice, the RNeasy spin column was moved to a new 1.5 mL collection tube, 30 μL of RNase-free water was added and centrifuged, and RNAs were eluted. RNA concentration was measured using the microRNA assay kit (Thermo Fisher Scientific, Waltham, MA, USA) with Qubit 3.0 Fluorometr (Thermo Fisher Scientific, Waltham, MA, USA).

#### 4.6.3. RNA Fragmentation and Purification

Using 10 μL of RNA with rRNA removed with the Low Input RiboMinus Eukaryote System v2 (Thermo Fisher Scientific, Waltham, MA, USA: A15027), 1 μL of 10× RNase Ⅲ Reaction Buffer and 1 μL of RNase Ⅲ were added and incubated at 37 °C for 2 min, 20 μL of nuclease-free water was added and placed on ice. An amount of 5 μL of suspended nucleic acid binding beads and 90 μL of binding solution concentrate were mixed, and 30 μL of fragmented RNA was added. A total of 150 μL of 100% ethanol was added, suspended well and incubated at room temperature for 5 min. After adding 150 μL of wash solution to the beads separated with a magnetic stand and incubating at room temperature for 30 s, the beads were separated again and allowed to dry naturally. After adding 12 μL of nuclease-free water at 37 °C, mixing the beads, and incubating at room temperature for 1 min, the beads were separated and the eluate was collected.

#### 4.6.4. Construction of Whole Transcriptome Library and Reverse Transcription Reaction

After adding 5 μL of hybridization master mix adjusted on ice to 3 μL of fragmented RNA and mixing them, adapter hybridization was performed (at 65 °C for 10 min, at 30 °C for 5 min). RNA ligation reagents (2X Ligation Buffer 10 μL, Ligation Enzyme Mix 2 μL) were added to this, and ligation reaction was performed at 30 °C for 1 h. Reverse transcription (RT) master mix was adjusted on ice (total 16 μL), mixed with 20 μL of RNA, and incubated at 70 °C for 10 min. A total of 4 μL of 10× SuperScript Ⅲ Enzyme Mix was added, and reverse transcription reaction was performed at 42 °C for 30 min.

#### 4.6.5. cDNA Purification

Furthermore, 5 μL of suspended nucleic acid binding beads and 120 μL of binding solution concentrate were mixed. In addition, 60 μL of nuclease-free water was added to 40 μL of the solution after reverse transcription reaction, mixed with the bead solution, and 125 μL of 100% ethanol was added. After incubating at room temperature for 5 min to bind the cDNAs to the beads, the cDNA eluate was collected as in the fragmented RNA purification procedure.

#### 4.6.6. cDNA Amplification, Purification and Library Quantification

Platinum PCR SuperMix High Fidelity (45 μL) and barcode primers at 1 μL each were adjusted, 6 μL of cDNA was added, and amplification reaction was performed (a 2-min hold at 94 °C. Two cycles for the following: 30 s at 94 °C, 30 s at 50 °C, 30 s at 68 °C. Sixteen cycles for the following: 30 s at 94 °C, 30 s at 62 °C, 30 s at 68 °C, and a 5-min hold at 68 °C). Additionally, 5 μL of suspended nucleic acid binding beads and 120 μL of binding solution concentrate were mixed, and 53 μL of amplified cDNA was added. After adding 130 μL of 100% ethanol, mixing well, incubating at room temperature for 5 min and binding cDNAs to the beads, the cDNA eluate was collected as in the fragmented RNA purification procedure (However, 15 μL of nuclease-free water was added). Using Agilent 4150 TapeStation (Agilent Technologies, Santa Clara, CA, USA), library quantification was performed. In addition, the reagents used were as follows:

- Ion Total RNA-Seq Kit v2 (Thermo Fisher Scientific, USA: 4475936);
- IonXpress RNA-Seq Barcode 1–16 Kit (Thermo Fisher Scientific, USA: 4475485);
- D1000 ScreenTape (Agilent Technologies, USA: 5067–5582);
- D1000 Reagents (Agilent Technologies, USA: 5067–5583).

#### 4.6.7. Sequencing

Using the adjusted libraries, next-generation sequencing was performed. The device used was Ion GeneStudio S5 (Thermo Fisher Scientific, Waltham, MA, USA), and the reagents used were Ion 540 Kit-OT2 and Ion 540 Chip (Thermo Fisher Scientific, Waltham, MA, USA: A27753, A27765).

#### 4.6.8. Expression Analysis

The sequence read of each sample was aligned against the rat reference (rn7, UCSC) using STAR (ver. 2.7.9a), and the expression was quantified with RSEM (ver. 1.3.3). Differential expression analysis was performed on the combination of two groups with DESeq2 (ver. 1.30.1).

### 4.7. Examination of Cell Proliferative Ability

EVs were extracted from bile 24 h after PH and after sham surgery with the abovementioned methods using the qEV original (35 nm) extracellular vesicle extraction kit. The rat cell line (BRL-3A) inoculated to a 96-well plate at 1 × 10^4^ cells/well was cultured under 5% CO_2_ at 37 °C for 12 h (the medium was 200 μL each of: DMEM, 10% FBS, 1% PS). After removing the medium and washing once with PBS, 200 μL of exosome-free medium (DMEM, 10% exosome-depleted FBS, 1% PS) was added, divided into the three groups of the PH-24 group, the sham group and the control group, and the PH-24 group and the sham group were further divided into two, with EVs added at 1.0 × 10^8^ particles/well to one, and at 1.0 × 10^9^ particles/well to the other. An extract without EVs (PBS) was added to the control group (*n* = 5 × MTS assay for four times).

After the addition of EVs, the MTS assay was performed according to the protocol using the MTS assay kit (Abcam, Cambridge, UK). MTS reagents were added by 20 μL to each well, and incubated under 5% CO_2_ at 37 °C for 1 h. Afterwards, using the ELISA plate reader (Benchmark Plus Microplate Spectrophotometer, Bio-Rad Laboratories Inc., Hercules, CA, USA), absorbance was measured at 490 nm. After culture under 5% CO_2_ at 37 °C for 24, 48, and 72 h, the MTS assay was similarly performed, and absorbance was measured.

### 4.8. Statistical Methods

Student’s *t*-test was used for the quantitative data comparison between two groups, and one-way ANOVA (Bonferroni’s multiple comparison) was used for the comparison between multiple groups. The *p* value < 0.05 was set as statistically significant. All statistical analysis was carried out using R software.

Gene ontology (GO) enrichment analysis was performed for transcriptome analysis (data = results of comparison between the PH-24 group and the control group, the sham group and the control group). For two-group comparison results, adjusted *p* value < 0.05 and Log_2_ fold change > 1 were regarded as upregulation, while adjusted *p* value < 0.05 and Log_2_ fold change < −1 were regarded as downregulation. Using these two-group comparison results, GO analysis was performed with the clusterProfiler R package (ver. 3.18.1). Analysis conditions were as follows:

- ontology = Biological Process;
- *p*-value cutoff = 0.5;
- p.adjust.method = FDR.

The results were narrowed down to those with *p* value < 0.01 and containing GO term = cell cycle, and the expression levels (quantitative results) of upregulated genes and downregulated genes were compared by PH-24 vs. sham using a *t*-test (*p* value < 0.05 was set as statistically significant).

### 4.9. Ethical Considerations

This study was an animal experiment using rats. The study plan was drafted in compliance with regulations and guidelines related to animal experiments, and was approved by the Yamagata University Animal Experiment Committee. As the category of the assumed pain is D (experiments using vertebrates that may be accompanied by inevitable severe stress or pain), anesthetics/analgesics were used to alleviate pain. In addition, humanitarian endpoints were set, and experiment animals were euthanized at an appropriate time when experiencing unbearable pain.

## 5. Conclusions

In this study, it was clarified that bile EVs increase after PH, PH24-EVs promote the proliferation of hepatocytes when added to the hepatocyte line, and genes promoting cell cycles are upregulated in hepatocytes. In the future, clinical application of newly discovered liver regeneration mechanisms by bile EVs may be anticipated.

## Figures and Tables

**Table 1:** Gene ontology (GO) enrichment analysis using the expression data of the PH-24 group and the sham group compared with the control group. When narrowing down by GO term containing “cell cycle” and *p* value < 0.01, three types of GO terms were obtained in the PH-24 group. *p* < 0.01 (**).

ID	Term	GeneRatio	*p* Value	*p* Adjusted	Count
GO:0044772	mitotic cell cycle phase transition	122/4596	0.0012 **	0.0755	122
GO:0044770	cell cycle phase transition	133/4596	0.0015 **	0.0823	133
GO:0044843	cell cycle G1/S phase transition	67/4596	0.0041 **	0.1334	67

**Table 2:** Upregulated and downregulated genes in the PH-24 group compared to the sham group (extracting those with *p* value < 0.05). *p* < 0.05 (*), *p* < 0.01 (**), *p* < 0.001 (***).

Up Regulated				Down Regulated			
Gene Symbol	Log2 Fold Change		*p* Value	Gene Symbol	Log2 Fold Change		*p* Value
	PH (24 h)	Sham			PH (24 h)	Sham	
Hacd1	1.0618	−0.5067	<0.0001 ***	Pidd1	−7.2416	0.0024	<0.0001 ***
Rptor	1.2947	0.1998	0.0003 ***	Rcc1	−1.1955	−0.1498	0.0001 ***
Rpl17	1.6784	−0.3205	0.0008 ***	Cirbp	−1.0556	−0.0983	0.0015 **
Tmod3	1.3730	0.1943	0.0009 ***	Plcb1	−6.8962	0.2994	0.0032 **
Chmp4c	2.4938	1.0174	0.0010 **	Camk2b	−5.5836	0.6711	0.0055 **
E2f3	3.6683	1.1763	0.0010 **	LOC102555319	−6.4815	−0.0902	0.0081 **
Bcl2	1.0480	0.1521	0.0010 **	Ccnj	−7.3298	0.0335	0.0099 **
Ccni	2.3697	0.9410	0.0013 **	Inhba	−6.4521	0.4295	0.0139 *
Vps4a	1.1111	0.1982	0.0016 **	Eps8	−6.2677	−0.2896	0.0204 *
Ctdsp2	1.3427	0.4218	0.0016 **	Hus1	−6.4833	−0.0456	0.0210 *
Anxa1	1.4453	0.5099	0.0028 **	Trim39	−6.7484	−0.2955	0.0217 *
Brsk1	3.3370	1.0763	0.0031 **	Fbxl6	−1.3573	−0.3980	0.0281 *
Cit	2.1396	0.6820	0.0031 **	Haspin	−1.0681	−0.3645	0.0318 *
Chek2	1.8543	0.5042	0.0034 **	Eif4e	−1.0115	−0.3972	0.0355 *
Dbf4	2.0209	0.8112	0.0035 **	Appl2	−6.8909	−0.3617	0.0366 *
Ppm1d	2.0957	0.7411	0.0041 **	Xrcc3	−6.7349	−0.4998	0.0371 *
Mad2l1	1.4244	0.2698	0.0060 **	Cdk5rap3	−1.0748	−0.3954	0.0395 *
Donson	1.8242	0.6657	0.0123 *	Foxm1	−1.5041	−0.2065	0.0461 *
Ddx3x	1.8009	0.9050	0.0146 *	Pdpn	−1.7263	−0.5369	0.0473 *
Pias1	1.4560	0.5660	0.0163 *				
Sox4	1.6782	0.7718	0.0217 *				
Adamts1	1.2637	0.2707	0.0225 *				
Pten	1.0504	0.5068	0.0278 *				
Gen1	1.4655	0.5676	0.0300 *				
Nek6	1.3541	0.6354	0.0316 *				
Mnat1	1.1033	0.6977	0.0347 *				
Mastl	1.9228	1.1178	0.0449 *				
Dcun1d3	1.1699	0.1316	0.0476 *				

## Data Availability

Not applicable.
